# The systemic and hepatic alternative renin–angiotensin system is activated in liver cirrhosis, linked to endothelial dysfunction and inflammation

**DOI:** 10.1038/s41598-023-28239-2

**Published:** 2023-01-18

**Authors:** Lukas Hartl, Benedikt Rumpf, Oliver Domenig, Benedikt Simbrunner, Rafael Paternostro, Mathias Jachs, Marko Poglitsch, Rodrig Marculescu, Michael Trauner, Roman Reindl-Schwaighofer, Manfred Hecking, Mattias Mandorfer, Thomas Reiberger

**Affiliations:** 1 Division of Gastroenterology and Hepatology, Department of Medicine III, Medical University of Vienna, Waehringer Guertel 18-20, 1090 Vienna, Austria; 2 Vienna Hepatic Hemodynamic Lab, Division of Gastroenterology and Hepatology, Department of Medicine III, Medical University of Vienna, Vienna, Austria; 3 Department of Surgery, Medical University of Vienna, Vienna, Austria; 4 Attoquant Diagnostics, Vienna, Austria; 5 Christian Doppler Lab for Portal Hypertension and Liver Fibrosis, Medical University of Vienna, Vienna, Austria; 6 Department for Laboratory Medicine, Medical University of Vienna, Vienna, Austria; 7 Division of Nephrology and Dialysis, Department of Medicine III, Medical University of Vienna, Vienna, Austria

**Keywords:** Portal hypertension, Ascites, Liver

## Abstract

We aimed to assess the systemic and hepatic renin-angiotensin-system (RAS) fingerprint in advanced chronic liver disease (ACLD). This prospective study included 13 compensated (cACLD) and 12 decompensated ACLD (dACLD) patients undergoing hepatic venous pressure gradient (HVPG) measurement. Plasma components (all patients) and liver-local enzymes (n = 5) of the RAS were analyzed using liquid chromatography–tandem mass spectrometry. Patients with dACLD had significantly higher angiotensin (Ang) I, Ang II and aldosterone plasma levels. Ang 1–7, a major mediator of the alternative RAS, was almost exclusively detectable in dACLD (n = 12/13; vs. n = 1/13 in cACLD). Also, dACLD patients had higher Ang 1–5 (33.5 pmol/L versus cACLD: 6.6 pmol/L, p < 0.001) and numerically higher Ang III and Ang IV levels. Ang 1–7 correlated with HVPG (ρ = 0.655; p < 0.001), von Willebrand Factor (ρ = 0.681; p < 0.001), MELD (ρ = 0.593; p = 0.002) and interleukin-6 (ρ = 0.418; p = 0.047). Considerable activity of ACE, chymase, ACE2, and neprilysin was detectable in all liver biopsies, with highest chymase and ACE2 activity in cACLD patients. While liver-local classical and alternative RAS activity was already observed in cACLD, systemic activation of alternative RAS components occurred only in dACLD. Increased Ang 1–7 was linked to severe liver disease, portal hypertension, endothelial dysfunction and inflammation.

## Introduction

While advanced chronic liver disease (ACLD) is initially asymptomatic and therefore considered compensated (cACLD), it eventually progresses into decompensated disease (dACLD), displaying characteristic symptoms which arise from portal hypertension such as ascites and bleeding of oesophageal varices^1,2^. Aside from symptom-dependent treatment, the current therapies of dACLD focus on lowering portal pressure and include pharmacological treatment using non-selective betablockers^3,4^ as well as invasive procedures such as the implantation of a transjugular intrahepatic portosystemic shunt (TIPS)^5^.

A better understanding of the role of the renin-angiotensin-system (RAS) including its classical and alternative components in ACLD may reveal novel therapeutic targets. Through renin cleaving hepatic angiotensinogen, angiotensin I (Ang I) is created and further converted to angiotensin (Ang) II by angiotensin-converting enzyme (ACE). Ang II is also frequently converted by the locally active chymase^6^ and mediates its effects through binding to members of the angiotensin-receptor family, which lead to vasoconstriction and aldosterone secretion facilitating sodium and water retention in the kidneys as well as mediating inflammation^7^. By acting not only systemically but also locally, the RAS is involved in growth and remodeling processes in the heart and blood vessels^8^. A permanently upregulated RAS is strongly associated with various health complications such as hypertension, diabetes and aging processes^9^. In liver disease, elevated Ang II is associated with both hepatic resistance as well as portal pressure^10^. Additionally, an increased expression of RAS components has even been observed in activated hepatic stellate cells (HSC) following liver injury, which enables local synthesis of Ang II mediating fibrosis of the liver^11,12^.

As opposed to the classical RAS-axis, the metalloprotease angiotensin-converting enzyme 2 (ACE2) has been identified as the key enzyme of the alternative RAS. It is able to use Ang II as substrate, converting it into Angiotensin 1–7 (Ang 1–7)^7^, which binds to the mas-receptor (masR) and has vasodilative and anti-inflammatory qualities^13^.

Ang 1–7 can also be formed by way of conversion through neprilysin (NEP) from Ang I. Upregulated Ang 1–7 is associated with lower incidence of steatosis^14^ and even plays a role in the inhibition of Ang II-mediated pathways facilitating fibrosis in cultured HSC^15^. Hence it can be argued, that ACE2 and Ang 1–7 as part of the alternate RAS axis act as a compensating measure to the classical RAS in the liver.

Considering the growing body of evidence on the involvement of RAS in liver disease, it seems essential to systematically investigate the classical and non-classical RAS fingerprint in patients across the full spectrum of liver disease. Thus, we performed a study to assess the blood and hepatic fingerprint of the classical and non-classical RAS components in the systemic (blood) and local (liver) compartments in patients with both compensated and decompensated ACLD.

## Results

### Patient characteristics (Table 1)

Overall, 25 patients (cACLD: n = 13, dACLD: n = 12) with a median age of 61.1 years and male predominance (n = 18/25; 72.0%) were included. Etiologies of liver disease included alcoholic liver disease (ALD; n = 10/25; 40.0%), viral hepatitis (n = 9/25; 36.0%), NASH (n = 4/25; 16.0%) and cholestatic liver disease (n = 2; 8.0%).

**Table 1:** Patient characteristics of patients with compensated (cACLD) and decompensated cirrhosis (dACLD).

Patient characteristics	cACLD (n = 13)	dACLD (n = 12)	p-value
Sex, male/female (% male)	11/2 (84.6%)	7/5 (58.3%)	0.144
Age, years (IQR)	59.3 (13.5)	62.5 (19.8)	0.999
Body mass index, kg m^−2^ (IQR)	24.6 (5.7)	28.3 (9.4)	0.017
Etiology			0.227
ALD, n (%)	3 (23.1%)	7 (58.3%)	
Viral hepatitis, n (%)	7 (53.8%)	2 (16.7%)	
NASH, n (%)	2 (15.4%)	2 (16.7%)	
Cholestatic, n (%)	1 (7.7%)	1 (8.3%)	
Varices, n (%)	3 (23.1%)	11 (91.7%)	< 0.001
History of variceal bleeding, n (%)	0 (0.0%)	4 (33.3%)	0.023
Ascites, n (%)	0 (0.0%)	12 (100.0%)	< 0.001
Refractory ascites, n (%)	0 (0.0%)	6 (50.0%)	0.003
History of spontaneous bacterial peritonitis	0 (0.0%)	1 (8.3%)	0.288
Hepatic encephalopathy, n (%)	0 (0.0%)	8 (66.7%)	0.003
Child–Turcotte–Pugh Score, points (IQR)	5.0 (0.0)	8.0 (3.0)	< 0.001
Child-stage			< 0.001
A, n (%)	13 (100.0%)	0 (0.0%)	
B, n (%)	0 (0.0%)	9 (75.0%)	
C, n (%)	0 (0.0%)	3 (25.0%)	
MELD, points (IQR)	9.0 (2.5)	15.5 (10.3)	< 0.001
Hepatic venous pressure gradient, mmHg (IQR)	8.0 (3.0)	19.0 (10.0)	< 0.001
Liver stiffness measurement, kPa (IQR)	13.8 (6.2)	62.4 (44.6)	< 0.001
Mean arterial pressure, mmHg (IQR)	98.0 (17.8)	96.0 (29.1)	0.999
Sodium, mmol L^−1^ (IQR)	140.0 (5.0)	134.0 (5.0)	0.015
Bilirubin, mg dL^−1^ (IQR)	0.8 (0.8)	1.2 (1.2)	0.115
International normalized ratio, units (IQR)	1.2 (0.2)	1.4 (0.6)	0.036
Albumin, g L^−1^ (IQR)	40.4 (4.1)	31.6 (5.8)	0.005
Creatinine, mg dL^−1^ (IQR)	0.8 (0.3)	1.0 (1.0)	0.434
VWF antigen, % (IQR)	242.0 (155.0)	413.0 (24.0)	< 0.001
C-reactive protein, mg dL^−1^ (IQR)	0.1 (0.3)	0.5 (1.9)	0.434
Interleukin-6, pg mL^−1^ (IQR)	4.0 (18.2)	22.2 (19.0)	0.100

*cACLD* compensated advanced chronic liver disease, *ALD* alcoholic liver disease, *dACLD* decompensated advanced chronic liver disease, *IQR* interquartile range, *MELD* model for end-stage liver disease, *NASH *non-alcoholic steatohepatitis, *VWF* von Willebrand factor.

A detailed comparison of the characteristics of patients with cACLD and dACLD is given in Table 1. In short, there was no difference in sex, median age or liver disease etiology between patients with cACLD and dACLD. Patients with cACLD had lower prevalence of varices, lower MELD score, HVPG, LSM, as well as higher plasma sodium and plasma albumin levels as compared to patients with dACLD.

Interestingly, median MAP did not differ between the cACLD and the dACLD cohort (cACLD: 98.0 mmHg vs. dACLD: 96.0 mmHg; p = 0.999).

VWF antigen (cACLD: 242.0% vs. dACLD: 413.0%; p < 0.001) and in tendency IL-6 levels (cACLD: 4.0 pg/mL vs. dACLD: 22.2 pg/mL; p = 0.100) were lower in patients with cACLD than in patients with dACLD, while CRP was not significantly different(cACLD: 0.1 mg/dL vs. dACLD: 0.5 mg/dL; p = 0.434).

### Plasma equilibrium levels of classical and alternative RAS components in patients with cACLD and dACLD (Table 2, Fig. 1)

**Figure 1:**
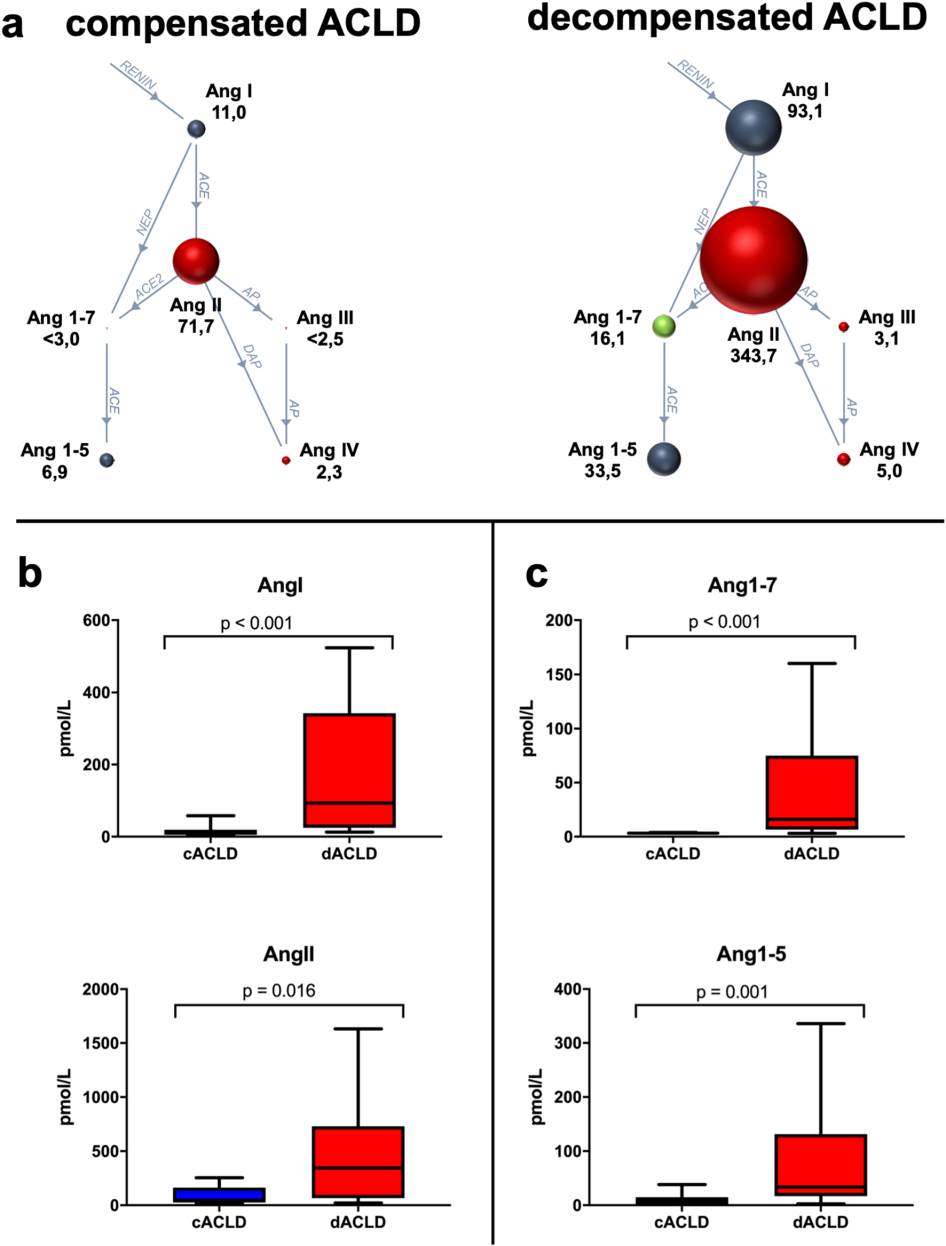
Components of the classical and alternative renin–angiotensin system (RAS) in patients with compensated (cACLD, n = 13) and decompensated cirrhosis (dACLD, n = 12). (**a**) Depiction of the RAS fingerprint in patients with cACLD and dACLD. Comparison of quantitative levels of (**b**) classical RAS components (Ang I and Ang II) and (**c**) alternative RAS components (Ang 1–7, Ang 1–5) between patients with cACLD and dACLD. For RAS fingerprint analysis, the size of the spheres and numbers beside them represent the median absolute concentrations of angiotensins (pmol/L) analyzed by mass spectrometry. *Ang* angiotensin, *ACE* angiotensin converting enzyme, *AP* aminopeptidase, *cACLD* compensated advanced chronic liver disease, *dACLD* decompensated advanced chronic liver disease, *DAP* dipeptidyl aminopeptidase, *NEP* neprilysin, *RAS* renin–angiotensin system.

Figure 1A shows the systemic RAS fingerprint in patients with cACLD and dACLD. The RAS was generally upregulated in dACLD with high levels of Ang I, Ang II, but also Ang 1–7 and Ang 1–5. RAS activity in the plasma of patients with cACLD was less pronounced. Specifically, PRA-S (cACLD: 51.5 pmol/L vs. dACLD: 489.7 pmol/L; p = 0.001), Ang I (cACLD: 12.4 pmol/L vs. dACLD: 93.1 pmol/L; p < 0.001), Ang II (cACLD: 46.2 pmol/L vs. dACLD: 343.7 pmol/L; p = 0.016) and aldosterone (cACLD: 84.0 pg/mL vs. dACLD: 383.6 pg/mL; p = 0.007) were lower in patients with cACLD, in comparison to patients with dACLD.

**Table 2:** Plasma levels of classical and alternative renin-angiotensin system (RAS) components in patients with compensated (cACLD) and decompensated cirrhosis (dACLD).

Parameter	cACLD (n = 13)	dACLD (n = 12)	p-value
PRA-S, pmol L^−1^ (IQR)	51.5 (150.0)	489.7 (710.1)	0.001
ACE-S, (pmol L^−1^) × (pmol L^−1^) (IQR)	6.6 (4.4)	3.4 (2.5)	0.022
Ang I, pmol L^−1^ (IQR)	12.4 (14.4)	93.1 (317.8)	< 0.001
Ang II, pmol L^−1^ (IQR)	46.2 (136.4)	343.7 (666.4)	0.016
Ang 1–7, pmo L^−1^ (IQR)	3.0 (0.0)	16.1 (68.3)	< 0.001
Undetectable Ang 1–7, n (%)	12 (92.3%)	2 (16.7%)	< 0.001
Ang 1–5, pmol L^−1^ (IQR)	6.6 (12.8)	33.5 (114.5)	< 0.001
Ang III, pmol L^−1^ (IQR)	2.5 (0.9)	3.1 (10.5)	0.309
Ang IV, pmol L^−1^ (IQR)	2.0 (2.8)	5.0 (16.6)	0.141
Aldosterone, pg mL^−1^ (IQR)^a^	84.0 (100.7)	383.6 (499.0)	0.007

*ACE-S* angiotensin converting enzyme surrogate, *Ang* angiotensin, *cACLD* compensated advanced chronic liver disease,* dACLD* decompensated advanced chronic liver disease, *IQR* interquartile range, *PRA-S* plasma renin activity surrogate, *RAS* renin–angiotensin system.

^a^Available in n = 14 patients (cACLD: n = 7; dACLD: n = 7).

Ang 1–7, a key component of the alternative RAS, was below the lower limit of quantification (i.e. < 3 pmol/L) in 92.3% (n = 12/13) of patients with cACLD, while in 83.3% (n = 10/12) of patients with dACLD patients, Ang 1–7 was detectable at a median level of 16.1 pmol/L (vs. 3.0 pmol/L in the one patient with cACLD; p < 0.001). Similarly, Ang 1–5 levels were lower in patients with cACLD than patients with dACLD (cACLD: 6.6 pmol/L vs. dACLD: 33.5 pmol/L; p < 0.001).

There was no significant difference in plasma levels of Ang III and Ang IV between patients with cACLD and patients with dACLD (Table 2).

### Correlations of RAS components and severity of liver disease, portal hypertension, liver stiffness, endothelial dysfunction and inflammation (Table S1, Fig. 2)

We determined associations between components of the classical (PRA-S, Ang I, Ang II) and alternative RAS (Ang 1–7) and parameters of liver disease severity (i.e. MELD), LSM, portal hypertension (i.e. HVPG), endothelial dysfunction (i.e. VWF antigen) and inflammation (i.e. IL-6).

**Figure 2:**
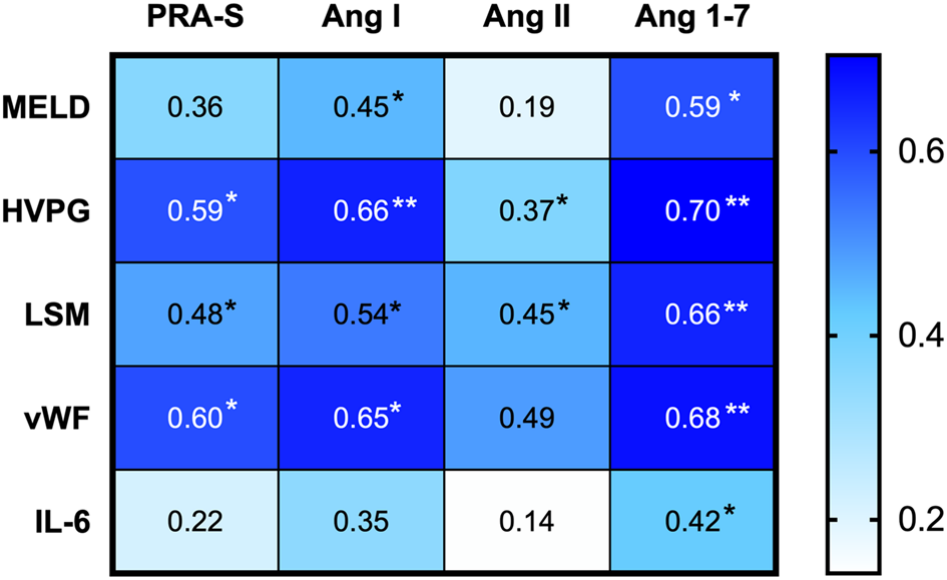
Correlations between components of the classical and alternative RAS (PRA-S, Ang I, Ang II and Ang 1–7) and parameters of liver disease severity (MELD), severity of portal hypertension (HVPG), liver stiffness (LSM), endothelial dysfunction (vWF) and inflammation (IL-6). Correlations were assessed using Spearman’s Rho. ∗ denotes p values < 0.05, whereas ∗∗ indicates p values < 0.001. *Ang* angiotensin, *HVPG* hepatic venous pressure gradient, *IL-6* interleukin-6, *LSM* liver stiffness measurement, *MELD* model for end-stage liver disease, *RAS* renin–angiotensin system, *PRA-S* plasma renin activity surrogate, *vWF* von Willebrand factor antigen.

Importantly, Ang 1–7 showed the strongest correlations of all RAS components with all assessed parameters. Ang 1–7 levels correlated with LSM (ρ = 0.704; p < 0.001), HVPG (ρ = 0.655; p < 0.001), VWF antigen (ρ = 0.681; p < 0.001), MELD (ρ = 0.593; p = 0.002) and IL-6 (ρ = 0.418; p = 0.047).

PRA-S exhibited correlations with LSM (ρ = 0.479; p = 0.028), HVPG (ρ = 0.592; p = 0.002), VWF antigen (ρ = 0.598; p = 0.002) and in tendency with MELD score (ρ = 0.360; p = 0.078). Ang I correlated with MELD score (ρ = 0.449; p = 0.024), LSM (ρ = 0.536; p = 0.012), HVPG (ρ = 0.658; p < 0.001) and VWF antigen (ρ = 0.654; p = 0.001), while Ang II correlated with LSM (ρ = 0.455; p = 0.038), VWF antigen (ρ = 0.489; p = 0.015) and tendentially with HVPG (ρ = 0.371; p = 0.068).

Assessed by linear regression analysis, HVPG (HR 2.61; 95% confidence interval [95% CI]: 0.69–4.52; p = 0.010; Table 3), VWF (HR 0.15; 95% CI: 0.01–0.30; p = 0.049) and dACLD (HR 7.67; 95% CI: 1.65–13.70; p = 0.015) were associated with Ang 1–7. In multivariate analysis, HVPG remained as the only parameter independently linked to Ang 1–7 plasma levels (aHR 2.57; 95% CI: 0.60–4.54; p = 0.013).

**Table 3:** Factors associated with Ang 1–7 assessed by linear regression analysis. Both univariate and multivariate regression analyses are shown.

Parameters	Univariate model			Multivariate model		
	HR	95% CI	p-value	aHR	95% CI	p-value
Age, years	1.10	−0.61 to 2.81	0.195	–	–	–
Sex, male	14.7	−23.01 to 52.42	0.428	–	–	–
MELD, points	2.10	−0.78 to 5.00	0.145	–	–	–
HVPG, mmHg	2.61	0.69 to 4.52	0.010	2.57	0.60 to 4.54	0.013
VWF, %	0.15	0.01 to 0.30	0.049	0.01	−0.23 to 0.26	0.905
IL-6, pg mL^−1^	0.21	−0.89 to 1.31	0.697	–	–	–
dACLD, yes	7.67	1.65 to 13.70	0.015	2.96	−8.15 to 14.05	0.586

*Ang* angiotensin, *HVPG* hepatic venous pressure gradient, *IL-6* interleukin-6, *LSM* liver stiffness measurement, *MELD* model for end-stage liver disease,* vWF* von Willebrand factor antigen.

### Differences of RAS activity in the plasma and liver tissue of ACLD patients (Table 4, Fig. 3, Fig. S1)

In five patients (cACLD: n = 3; dACLD: n = 2), components of the classical and alternative RAS were assessed both in plasma and liver tissue, as shown in Table 3. Again, plasma levels of classical (PRA-S, Ang I, Ang II, aldosterone) and alternative RAS components (Ang 1–7, Ang 1–5) were particularly high in dACLD patients (Fig. S1).

**Table 4:** Hepatic and systemic RAS components in liver tissue samples and plasma samples of 5 ACLD patients (#1–5).

	#1	#2	#3	#4	#5
Patient characteristics					
Age, years	69	67	62	64	52
Body mass index, kg m^−2^	26.1	24.6	23.1	33.9	19.3
Etiology	NASH	VIRAL	ALD	NASH	ALD
cACLD/dACLD	cACLD	cACLD	cACLD	dACLD	dACLD
HVPG, mmHg	15	3	8	10	18
MAP, mmHg	112	94	106	119	123
CTP score	5	5	5	6	8
MELD	11	12	9	8	19
Liver tissue					
ACE activity	286.0	292.0	119.0	116.0	185.0
Chymase activity	6971.0	302.0	359.0	731.0	2237.0
NEP activity	59.0	669.0	304.0	403.0	86.0
ACE2 activity	186.0	80.0	54.0	130.0	110.0
Plasma					
PRA-S	28.6	137.4	37.2	147.2	446.7
Ang I (1–10)	12.4	18.2	7.9	49.2	151.8
Ang II (1–8)	16.1	119.2	29.3	98.0	295.0
Ang 1–7	3.0	3.0	3.0	6.0	23.9
Ang 1–5	2.0	7.4	6.6	27.9	114.0
Ang III (2–8)	7.6	22.1	3.0	5.1	23.3
Ang IV (3–8)	2.0	5.7	2.0	2.9	12.0
Aldosterone	43.3	158.7	66.9	383.6	1339.9

In the liver tissue, activity of ACE and chymase as enzymes of the classical RAS and ACE2 and NEP as enzymes of the non-classical RAS were assessed. In plasma, levels of classical (PRA-S, Ang I, Ang II, aldosterone) and non-classical RAS (Ang 1–7, Ang III, Ang IV, Ang 1–5) were quantified.

*ACE* angiotensin converting enzyme, *Ang* angiotensin, *cACLD* compensated advanced chronic liver disease, *CTP* Child–Turcotte–Pugh, *dACLD* decompensated advanced chronic liver disease, *HVPG* hepatic venous pressure gradient, *MAP* mean arterial pressure, *MELD* model for end-stage liver disease, *NEP* neprilysin, *PRA-S* plasma renin activity surrogate, *RAS* renin–angiotensin system.

**Figure 3:**
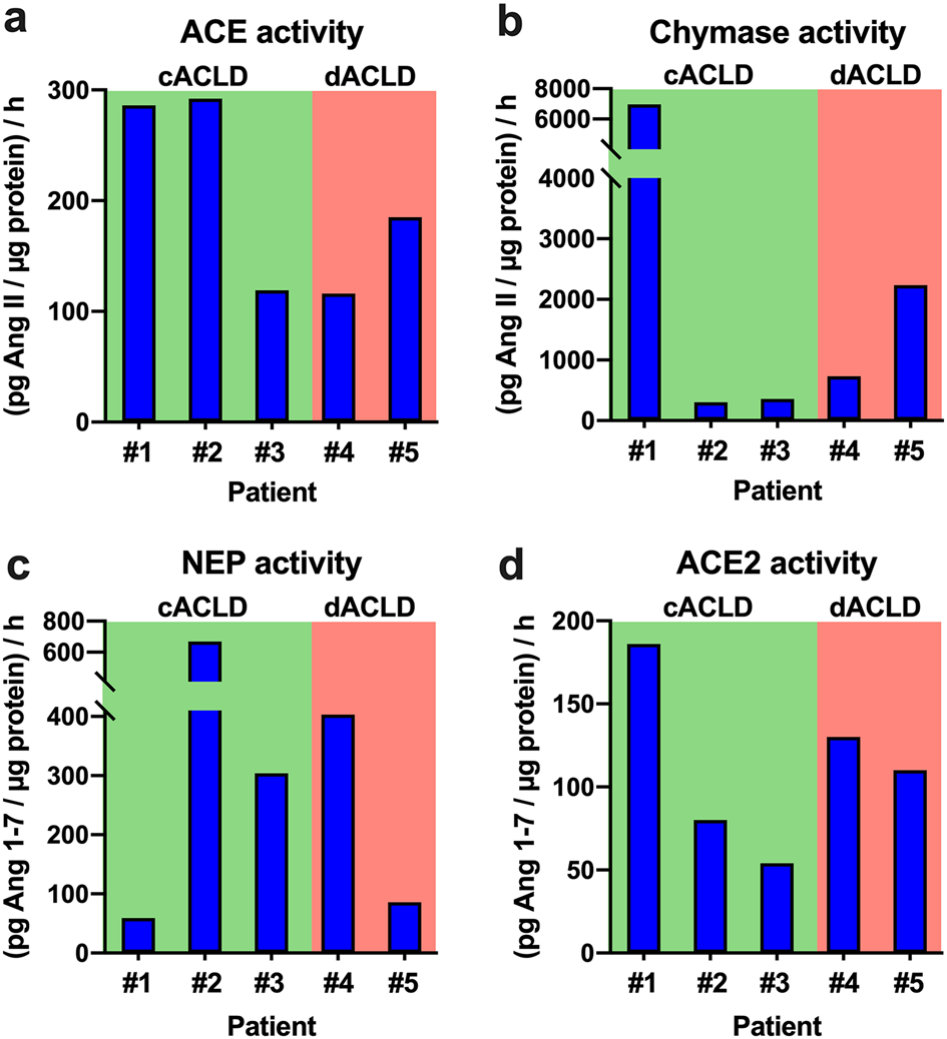
Tissue activity of components of the (**a**,**b**) classical (ACE, chymase) and (**c**,**d**) alternative RAS (neprilysin [NEP], ACE2) in 5 patients with compensated (cACLD; n = 3) and decompensated advanced chronic liver disease (dACLD; n = 2). *ACE* angiotensin converting enzyme, *cACLD* compensated advanced chronic liver disease, *dACLD* decompensated advanced chronic liver disease, *NEP* neprilysin, *RAS* renin–angiotensin system.

However, interestingly, the enzymatic activity of the RAS was more complex in the liver tissue, as outlined in Fig. 3.

Of note, patient #1 had compensated NASH and only moderate systemic classical or alternative RAS activity, and exhibited the highest hepatic activity of the classical RAS (ACE activity: 286.0 (pg Ang II/µg protein)/h; chymase activity: 6971.0 (pg Ang II/µg protein)/h) and ACE2 (186.0 (pg Ang 1–7/µg protein)/h), while NEP activity was the lowest among all patients (59.0 (pg Ang 1–7/µg protein)/h).

Patient #2 had viral hepatitis without portal hypertension (HVPG: 3 mmHg) and systemic levels moderate systemic levels of Ang II and Ang IV, while Ang I was low and Ang 1–7 was below the lower limit of quantification. This patient exhibited the highest hepatic activity of ACE (292.0 (pg Ang II/µg protein)/h) and NEP (669.0 (pg Ang II/µg protein)/h).

Patient #3 had compensated ALD and subclinical portal hypertension and had been abstinent for one month before study inclusion. This patients had virtually no systemic activation of the classical or alternative RAS and exhibited low hepatic activity of both the classical (ACE activity: 119.0 (pg Ang II/µg protein)/h; chymase activity: 359.0 (pg Ang II/µg protein)/h) and alternative RAS (ACE2 activity: 54.0 (pg Ang 1–7/µg protein)/h; NEP activity: 304.0 (pg Ang 1–7/µg protein)/h).

Patient #4 had decompensated NASH and relatively low systemic RAS activity, and exhibited moderate and high hepatic activity of the classical (ACE activity: 116.0 (pg Ang II/µg protein)/h; chymase activity: 731.0 (pg Ang II/µg protein)/h) and alternative RAS (ACE2 activity: 130.0 (pg Ang 1–7/µg protein)/h; NEP activity: 403.0 (pg Ang 1–7/µg protein)/h), respectively.

Patient #5, had decompensated ALD, high systemic classical and alternative RAS activation and exhibited pronounced hepatic activity of the classical RAS (ACE activity: 185.0 (pg Ang II/µg protein)/h; chymase activity: 2237.0 (pg Ang II/µg protein)/h). However, although systemic alternative RAS activity was high, hepatic activity of ACE2 (110.0 (pg Ang 1–7/µg protein)/h) and NEP (86.0 (pg Ang 1–7/µg protein)/h) were relatively low.

## Discussion

In this study, we assessed the classical and alternative RAS fingerprint in well-characterized patients with ACLD, divided into two cohorts: cACLD versus dACLD. We found that patients with dACLD had pronounced systemic activation of the classical RAS, and activation of the alternative RAS, Ang 1–7 being detectable in 83.3% of patients with dACLD (and in only one patient with cACLD [7.7%]). Importantly, we also demonstrated that, compared to other RAS components, Ang 1–7 showed the strongest correlations with parameters of liver disease severity, portal hypertension, LSM and endothelial dysfunction. Moreover, Ang 1–7 was the only angiotensin that correlated with IL-6 as a marker for systemic inflammation. Finally, suggest that the local activity of the classical and non-classical RAS in the liver is different from systemic RAS activity. Of note, patients with cACLD and patients with clinically significant portal hypertension exhibited the highest hepatic activities of classical and alternative RAS enzymes.

A systemic activation of the classical RAS in patients with dACLD, particularly in patients with ascites is well-documented^16–19^. In line with this, patients with dACLD in our cohort showed pronounced systemic elevation of classical RAS components including PRA-S, Ang I, Ang II and aldosterone. Higher classical RAS activity in dACLD aggravates hyperdynamic circulation, as it promotes splanchnic vasodilation and higher intrahepatic vascular resistance^19^. Moreover, ACE inhibition lowers portal pressure in cirrhotic patients^20–22^.

To date, data on alternative RAS activity in patients with ACLD are still scarce and evidence of alternative RAS activation in liver cirrhosis is mostly based on animal studies^19^. Our study shows that in the plasma of patients with dACLD, alternative RAS components including Ang 1–7 and Ang 1–5 were higher than in patients with cACLD. Similarly, activation of the alternative RAS was observed in diabetic patients with vascular disease and hypertension, in which ACE2 upregulation was interpreted as an attempt to counteract disease progression^23^. Whether this is the case in patients with dACLD or whether increased Ang 1–7 and Ang 1–5 are simply byproducts of overall RAS activation cannot be answered definitively with our data and requires further research.

At the same time, Ang III and Ang IV levels did not differ significantly between patients with cACLD and dACLD, but were numerically higher in patients who were decompensated. This result is in line with previous findings of a study displaying activation of the Ang 1–7/Mas receptor axis in 7 patients with liver cirrhosis undergoing liver transplantation^24^ and another study that showed increased levels of Ang 1–7 in the plasma of 9 cirrhotic patients and 23 non-cirrhotic patients with hepatitis C compared to healthy controls^25^. Moreover, several animal studies demonstrated systemic elevation of alternative RAS components including Ang 1–7 and ACE2 in chronic liver disease^26,27^.

Interestingly, previous studies suggest an activation of the systemic alternative RAS already in pre-cirrhotic stages of chronic liver disease, particularly in viral hepatitis^25^. In contrast, our cohort of patients who had strictly compensated ACLD of majorly viral etiology with mostly subclinical portal hypertension showed virtually no alternative RAS activity in their plasma, with Ang 1–7 being undetectable in 92.3% of patients in this cohort. This may be due to the fact that these were primarily patients after cure of chronic hepatitis C with correspondingly lower levels of (hepatic) inflammation.

Importantly, there were statistical correlations between Ang 1–7 and parameters of liver disease severity (MELD), LSM, portal hypertension (assessed by the gold standard HVPG) and endothelial dysfunction (assessed by its well-established surrogate biomarker VWF antigen^28–31^), suggesting a link between activation of the alternative RAS and worse outcomes of patients with ACLD^32,33^. Moreover, unlike other components of the RAS, Ang 1–7 correlated with IL-6, linking alternative RAS activation in ACLD to systemic inflammation^1^. Of note, systemic Ang II did not correlate with MELD or HVPG, indicating a special status of particularly the systemic alternative RAS in ACLD and portal hypertension. Interestingly, HVPG was the only parameter independently associated with Ang 1–7 in linear regression analysis, linking portal hypertension to alternative RAS activation.

An important role of the local intrahepatic classical RAS has been reported to drive an increase in portal hypertension^19,34^. Moreover, ACE inhibitors were associated with a decreased risk of liver-related events in non-alcoholic fatty liver disease^35^. There has been evidence that ACE2 is upregulated in livers of humans and rats^24,26,36^ with chronic liver disease. Furthermore, NEP was higher in livers of cirrhotic animals^37^. Our study, examining liver biopsies of 2 individuals with cACLD and 2 with dACLD, demonstrated that hepatic expression of enzymes of the classical and alternative RAS is complex and not limited to patients with dACLD. Despite exhibiting little classical and alternative RAS activity in their plasma, patients with cACLD had the highest hepatic activity of ACE, chymase, NEP and ACE2, suggesting a pronounced activation of intrahepatic classical and alternative RAS already in early stages of ACLD. This finding is of high relevance, as Ang II is known to induce and promote liver fibrosis via contraction, proliferation and activation of HSCs^11,38,39^ and hepatic chymase has also been linked to liver fibrosis in animals and humans^40,41^. Moreover, induction of intrahepatic ACE2 activity has been suggested to exert antifibrotic effects^42^ and NEP seems to be critically involved in the development of portal hypertension^37^ and kidney function in cirrhotic animal models^43^. Modifications such as ACE2 activation or Ang 1–7 supplementation seemed to have beneficial effects in murine models^44,45^.Thus, targeted pharmacological interventions targeting RAS components in cACLD patients may attenuate disease progression.

Our study also has limitations. Firstly, we recruited strictly compensated patients with cACLD, as well as patients with dACLD with a history of at least two decompensation events. Accordingly, this study does not investigate RAS activation patterns in other stages of ACLD. Secondly, this is a pilot study, including only a small number of patients with cACLD and dACLD. This limitation applies for the systemic (plasma) RAS fingerprint results and even more so for the intrahepatic RAS characterization (assessed in only 5 patients). Thus, the limited sample size represents an important limitation of our study and further studies are required to examine the systemic RAS fingerprint throughout all stages of ACLD, as well as the intrahepatic RAS in cACLD and dACLD. Still we want to emphasize that these data are novel and of important value for the liver community. Thirdly, collinearity between dACLD and other parameters, which were correlated with Ang 1–7, cannot be excluded, since these parameters increase with ACLD severity^1,46^. To account for this, linear regression analysis considering dACLD as a potential confounding factor was conducted. Also, patients with dACLD in our study still had relatively high median MAP, which indicates that patients with hyperdynamic circulation may be underrepresented in our study. Moreover, the majority of patients with dACLD had intake of aldosterone antagonists. This represents a confounding factor regarding the aldosterone levels reported in this study. Finally, plasma aldosterone levels were not available for all patients, but our results are well in line with previous studies^18,47^.

In conclusion, in this comprehensive characterization of the RAS fingerprint in patients with cACLD and dACLD, we were able to show that in addition to the classical RAS, the alternative RAS is activated in the plasma of those with dACLD. Importantly, Ang 1–7 correlated with parameters of liver disease severity, portal hypertension, endothelial dysfunction and inflammation. Hepatic activation of classical and alternative RAS components is complex and ACE, chymase, ACE2 and NEP are already upregulated in liver tissue of patients with cACLD.

## Methods

### Study population and sampling

This prospective single center study included patients who underwent hepatic venous pressure gradient (HVPG) measurement at the Vienna Hepatic Hemodynamic Lab (Division of Gastroenterology and Hepatology, Medical University of Vienna) between 06/2017 and 12/2021. ACLD was defined as liver stiffness measurement (LSM) ≥ 10 kPa and/or hepatic venous pressure gradient > 5 mmHg and/or histological fibrosis stage F3/F4. While exclusively strictly compensated ACLD patients in Child-stage A with without intake of diuretics or ACE inhibitors were evaluated for inclusion in the cACLD group, only clinically stable decompensated patients with ascites and another hepatic decompensation event (hepatic encephalopathy, refractory ascites, history of variceal bleeding, history of spontaneous bacterial peritonitis) were considered for the dACLD group. Patients with evidence of infection at the time of HVPG measurement, as well as patients with portal vein thrombosis, TIPS, hepatocellular carcinoma, or liver transplantation were excluded.

We aimed to measure components of both the classical and the alternate RAS from local and systemic sources. Liquid biopsies were collected as EDTA plasma through peripherally drawn venous blood samples. Liver tissue was collected by way of transjugular liver biopsy as previously described^48^. Both plasma and liver tissue samples were immediately stored at −80 °C.

### HVPG measurement

HVPG was measured according to a standardized procedure^48^. After local anesthesia, a catheter introducer sheath was placed in the right internal jugular vein. Using fluoroscopic guidance, a specifically designed angled-tip balloon catheter^49^ and was advanced into a large hepatic vein via the inferior vena cava. HVPG was calculated by subtracting free from wedged hepatic venous pressure. For further analyses, the mean of 3 measurements was used. Hemodynamic parameters including mean arterial pressure (MAP) were measured at the beginning of HVPG measurement.

### Assessment of liver stiffness measurement, liver function and endothelial dysfunction

Liver stiffness measurement (LSM) was measured via vibration-controlled transient elastography (VCTE) using Fibroscan© (Echosens, Paris, France). Severity of portal hypertension was evaluated by calculating the HVPG during liver vein catheterization. Serum von-Willebrand-factor was measured for evaluation of endothelial dysfunction and as an additional surrogate marker for portal pressure^33^. Child–Turcotte–Pugh (CTP) and Model for End stage Liver Disease score (MELD) of 2016^32^ were used to assess severity of ACLD.

### Laboratory analysis

Measurement of routine laboratory parameters such as interleukin-6 (IL-6) were performed by the facility’s department of laboratory medicine.

For assaying enzyme activities in tissue samples, liver tissue samples were homogenized under liquid nitrogen to enable stabilized peptide extraction and assayed as described previously^50^. Quantification of equilibrium RAS metabolites Ang I, Ang II, Ang III Ang IV as well as Ang 1–5 and Ang 1–7 in plasma was performed by liquid chromatography-tandem mass spectrometry (Attoquant Diagnostics, Vienna, Austria) using previously established protocols^51^. Plasma renin activity surrogate (PRA-S), a well-established biomarker for plasma renin activity was calculated as the sum of Ang I and Ang II^52,53^ and systemic ACE surrogate (ACE-S) was calculated as Ang II divided by Ang I^53,54^. Plasma aldosterone was assessed by chemiluminsescence immunoassay (DiaSorin, Liaision XL, Saluggia, Italy). Of note, plasma aldosterone levels were not available in all patients, as they were only measured starting 04/2018.

### Statistical analysis

Categorical variables were reported as number (n) of patients and % of patients with the characteristic of interest. Continuous data was presented as median and interquartile range (IQR). The data sets were tested for normal distribution with D’Agostino & Pearson and Shapiro–Wilk normality test. Mann–Whitney U test was used for comparing continuous variables without normal distribution between two groups and group comparisons of categorical variables were conducted using Pearson’s Chi-squared test. Correlations between two metric non-normally distributed variables were assessed with Spearman’s Rho. Linear regression analysis was conducted to investigate factors associated with Ang 1–7. Parameters that were at least tendentially associated with Ang 1–7 in univariate analysis (p < 0.100) were included in the multivariate model. GraphPad Prism 8 (Graphpad Software, La Jolla, CA, USA) and IBM SPSS 25.0 statistic software (IBM, Armonk, NY, USA) were used for statistical analysis. Two-sided p-values equal to or below 0.05 were considered as statistically significant.

### Ethics

All included patients were part of the Vienna Cirrhosis Study (VICIS; NCT: NCT03267615), a prospective observational trial. This study was performed in accordance with the current version of the Helsinki declaration and approved by the local Ethics Committee of the Medical University of Vienna (No. 1262/2017). All patients gave their informed written consent before study inclusion.

## Supplementary Information

Supplementary Information.

## Data Availability

The data is available upon reasonable request to the corresponding author.

## Abbreviations

95%CI: 95% Confidence interval
Ang: Angiotensin
ACE: Angiotensin converting enzyme
ACLD: Advanced chronic liver disease
ALD: Alcoholic liver disease
BMI: Body mass index
cACLD: Compensated advanced chronic liver disease
CSPH: Clinically significant portal hypertension
CTP: Child–Turcotte–Pugh
EC: Ethics committee
HVPG: Hepatic venous pressure gradient
HSC: Hepatic stellate cell
IQR: Interquartile range
IL-6: Interleukin-6
INR: International normalized ratio
LSM: Liver stiffness measurement
MAP: Mean arterial pressure
MELD: Model for end-stage liver disease
n: Number
NASH: Non-alcoholic steatohepatitis
NEP: Neprilysin
PRA-S: Plasma renin activity
RAS: Renin–aldosterone system
TIPS: Transjugular intrahepatic portosystemic shunt
VCTE: Vibration-controlled transient elastography
VWF: Von Willebrand factor
