# Supplementary material for: The systemic and hepatic alternative renin–angiotensin system is activated in liver cirrhosis, linked to endothelial dysfunction and inflammation
Source: Sci Rep. 2023 Jan 18;13:953. doi: 10.1038/s41598-023-28239-2 (PMC9849268; doi:10.1038/s41598-023-28239-2)

**SUPPLEMENTARY MATERIAL**

**The systemic and hepatic alternative renin-angiotensin system is activated in liver cirrhosis, linked to endothelial dysfunction and inflammation**

**Lukas HARTL, MD^1,2^ #,**

**Benedikt RUMPF, MD^1,3^ #,**

Oliver DOMENIG, PhD^4^ ,

Benedikt SIMBRUNNER, MD^1,2,5^ ,

Rafael PATERNOSTRO, MD^1,2^ ,

Mathias JACHS, MD^1,2^ ,

Marko POGLITSCH, PhD^4^ ,

Rodrig MARCULESCU, MD^6^ ,

Michael TRAUNER, MD^1^ ,

Roman REINDL-SCHWAIGHOFER, MD^7^ ,

Manfred HECKING, MD PhD^7^ ,

Mattias MANDORFER, MD PhD^1,2^ ,

Thomas REIBERGER, MD^1,2,5^ *

**# Authors contributed equally and share first authorship**

^1^ Division of Gastroenterology and Hepatology, Department of Medicine III, Medical University of Vienna, Vienna, Austria

^2^ Vienna Hepatic Hemodynamic Lab, Division of Gastroenterology and Hepatology, Department of Medicine III, Medical University of Vienna, Vienna, Austria

^3^ Department of Surgery, Medical University of Vienna, Vienna, Austria

^4^ Attoquant Diagnostics, Vienna, Austria

^5^ Christian Doppler Lab for Portal Hypertension and Liver Fibrosis, Medical University of Vienna, Vienna, Austria

^6^ Department for Laboratory Medicine, Medical University of Vienna, Vienna, Austria

^7^ Division of Nephrology and Dialysis, Department of Medicine III, Medical University of Vienna

**KEYWORDS**

**TABLE OF CONTENTS**

Table-S1. Correlations between components of the classical and non-classical RAS (plasma renin activity [PRA], Ang I, Ang II and Ang 1-7) and parameters of liver disease severity (MELD), severity of portal hypertension (HVPG), liver stiffness (LSM), endothelial dysfunction (vWF) and inflammation (IL-6).

Page 3

Figure-S1. Plasma levels of components of the classical (Ang I, Ang II) and alternative RAS (Ang 1-7, Ang IV) in 5 patients with compensated (cACLD; n=3) and decompensated advanced chronic liver disease (dACLD; n=2).

Page 4

**Table-S1. Correlations between components of the classical and non-classical RAS (plasma renin activity [PRA], Ang I, Ang II and Ang 1-7) and parameters of liver disease severity (MELD), severity of portal hypertension (HVPG), liver stiffness (LSM), endothelial dysfunction (vWF) and inflammation (IL-6).**

Correlations were assessed using Spearman’s Rho.

|  | **PRA** | | **Ang I** | | **Ang II** | | **Ang 1-7** | |
| --- | --- | --- | --- | --- | --- | --- | --- | --- |
|  | ρ | p-value | ρ | p-value | ρ | p-value | ρ | p-value |
| **MELD** | 0.360 | 0.078 | 0.449 | **0.024** | 0.192 | 0.358 | 0.593 | **0.002** |
| **HVPG** | 0.592 | **0.002** | 0.658 | **<0.001** | 0.371 | 0.068 | 0.704 | **<0.001** |
| **LSM** | 0.479 | **0.028** | 0.536 | **0.012** | 0.455 | **0.038** | 0.655 | **<0.001** |
| **VWF** | 0.598 | **0.002** | 0.654 | **0.001** | 0.489 | **0.015** | 0.681 | **<0.001** |
| **IL-6** | 0.219 | 0.316 | 0.346 | 0.106 | 0.142 | 0.519 | 0.418 | **0.047** |

**Figure-S1. Plasma levels of components of the classical (Ang I, Ang II) and alternative RAS (Ang 1-7, Ang IV) in 5 patients with compensated (cACLD; n=3) and decompensated advanced chronic liver disease (dACLD; n=2).**

*Abbreviations: Ang=angiotensin; cACLD=compensated advanced chronic liver disease; dACLD=decompensated advanced chronic liver disease; RAS=renin-angiotensin system*


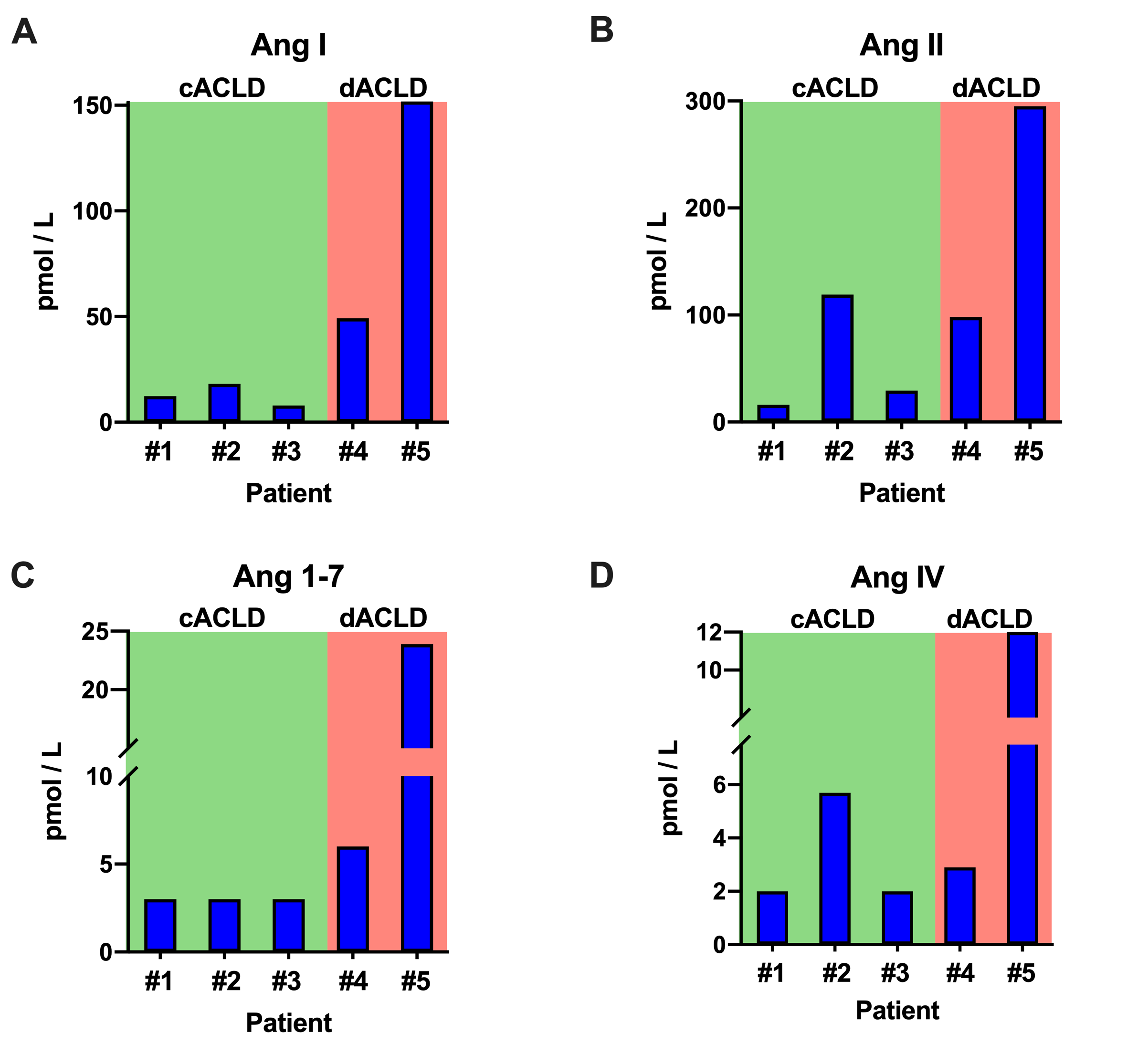

Supplement: Supplementary file 1 — Supplementary Information. [file 41598_2023_28239_MOESM1_ESM.docx]
